# Late presentation, MR imaging features and surgical treatment of Herlyn-Werner-Wunderlich syndrome (classification 2.2); a case report

**DOI:** 10.1186/s12905-018-0655-4

**Published:** 2018-10-03

**Authors:** Hidayatullah Hamidi, Nilab Haidary

**Affiliations:** 1 Radiology Department, French Medical Institute for Mothers and Children (FMIC), 3rd district, Kabul, Afghanistan; 2 Obstetrics and Gynecology Department, French Medical Institute for Mothers and Children (FMIC), Kabul, Afghanistan

**Keywords:** Herlyn-Werner-Wunderlich syndrome, Müllerian duct anomalies, Magnetic resonance imaging, Uterus didelphys, Blind hemivagina and ipsilateral renal agenesis

## Abstract

**Background:**

Herlyn-Werner-Wunderlich syndrome is a very rare congenital genitourinary anomaly characterized by uterus didelphys, blind hemivagina and ipsilateral renal agenesis.

**Case presentation:**

Authors present a case of Herlyn-Werner-Wunderlich syndrome in a 19-year-old unmarried woman who presented with pelvic pain and pelvic mass. MR imaging revealed the typical features of didelphys uterus, obstructed right hemivagina and ipsilateral renal agenesis. The patient subsequently underwent surgery.

**Conclusions:**

Herlyn-Werner-Wunderlich syndrome would be suspected in patients with unilateral absent kidney and pelvic mass. Ultrasonography and MR imaging can well depict the disease entity and surgery is the treatment of choice for obstructed hemivagina.

## Background

Herlyn-Werner-Wunderlich syndrome (HWWS) is name of a congenital anomaly characterized by uterus didelphys, blind hemivagina and ipsilateral renal agenesis [1] therefore it is also known as Obstructed hemivagina and Ipsilateral Renal Anomaly (OHVIRA) [2]. Authors present a case of HWWS in a 19-year-old unmarried woman presented with pelvic pain and pelvic mass. MR imaging revealed the typical features of didelphys uterus, obstructed right hemivagina and ipsilateral renal agenesis. The associated finding was right adnexal infected cyst. The patient subsequently underwent surgery.

## Case presentation

A 19-year-old, unmarried woman presented with complaint of chronic pelvic pain and a palpable mass at the lower pelvic midline region. On physical examination, a mobile and painless mass was palpated at the lower pelvic midline region. The external genitalia were normal in appearance. Transabdominal ultrasonography reported a cystic structure in the lower pelvic region communicating with the uterus (likely dilated vagina) with endometrial cavities and absent right kidney. A cystic structure with internal septae was also reported in the left adnexal region.

**Fig. 1:**
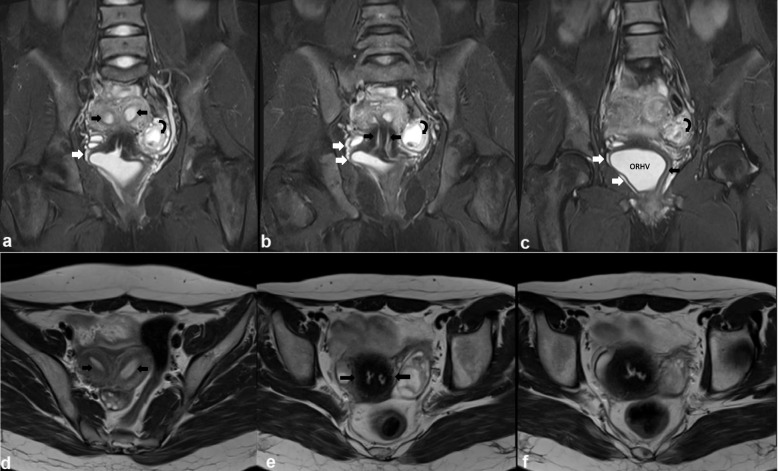
Coronal (**a**, **b** & **c**) and axial (**d**, **e** & **f**) T2WI images through the pelvis shows duplication of the endometrial canals, uterine cervices and vaginal canals (black arrows). The right vaginal canal is significantly dilated (c: ORHV). A small tubular structure with internal fluid signal along the anterolateral aspect of the dilated right hemivagina represented blind ectopic ureter (the mesonephric remnants) (white arrows). Left adnexal hemorrhagic cyst is also seen (curved arrow). There is communication between the two cervices well seen in axial section (**f**)

**Fig. 2:**
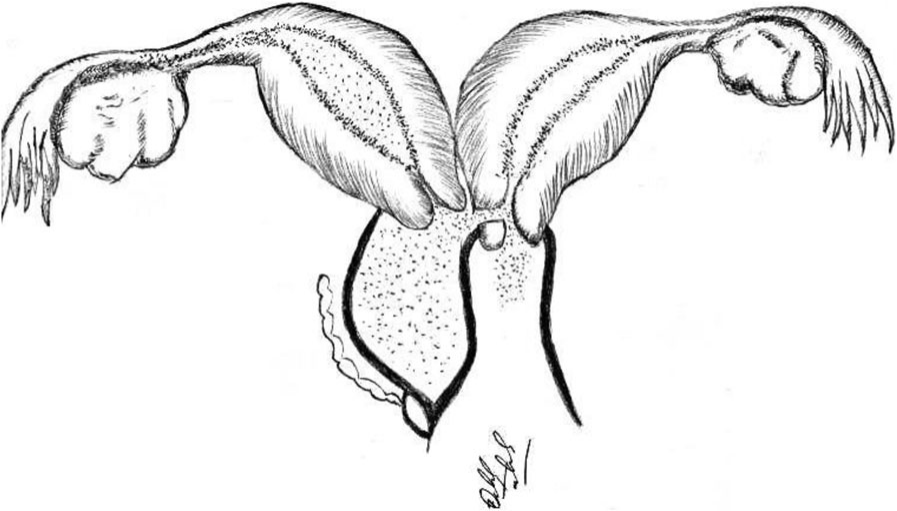
Schematic drawing of Herlyn-Werner-Wunderlich Syndrome (classification 2.2): duplication of the uterine bodies, endometrial canals, uterine cervices and vaginal canals, communication between two cervices, dilated right hemivagina and blinded ectopic right ureter (the mesonephric remnants). (Image drawn by: Habibullah)

**Fig. 3:**
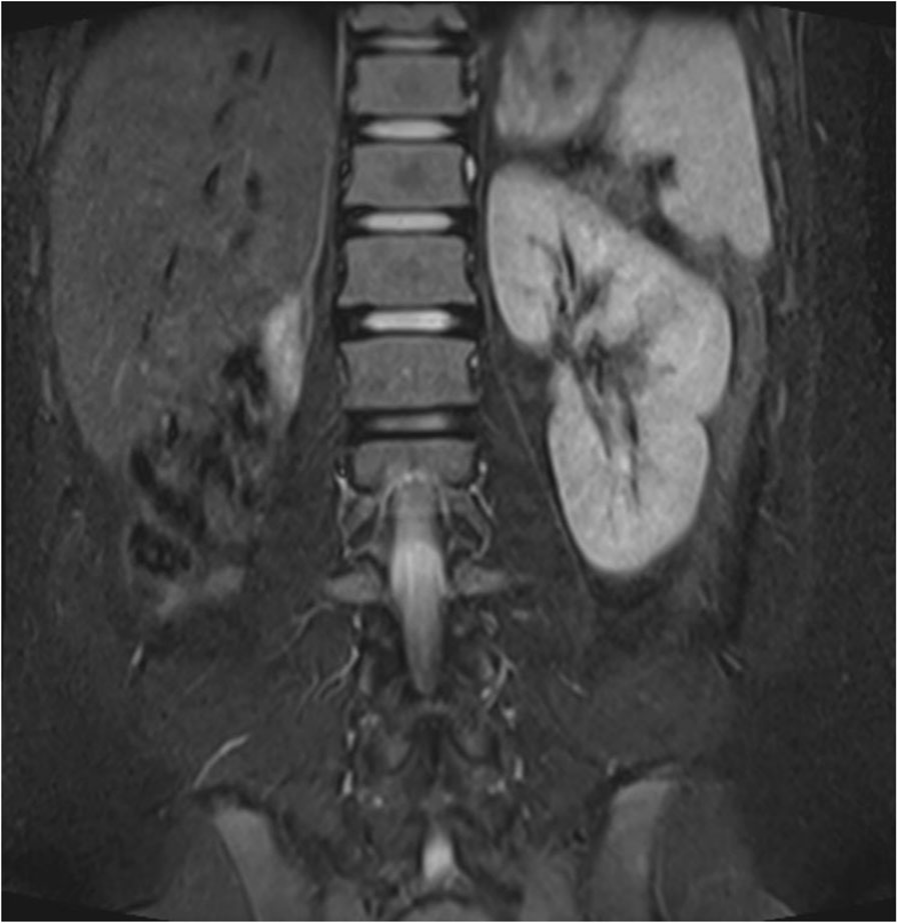
Coronal T2WI, right kidney is absent

MR imaging with contrast was performed and showed duplication of the uterine bodies, endometrial canals, uterine cervices and vaginal canals. The right vaginal canal was significantly dilated. There was communication between the two cervices well seen in the axial T2W sequence. A small tubular structure with internal fluid signal along the anterolateral aspect of the dilated right hemivagina represented blind ectopic ureter (the mesonephric remnants) (Figs. 1 and 2). Right kidney was not visualized in included sections of the upper abdomen (Fig. 3).

A heterogeneous cystic structure was seen in the left ovary with hemorrhagic components.

The patient underwent surgery under general anesthesia. The septum was excised and hematocolpos was drained. The hemorrhagic, infected left adnexal cyst was also excised.

## Discussion and conclusions

HWWS is a very rare congenital anomaly [3]. It represents a type of Müllerian duct anomalies associated with mesonephric duct anomalies exhibited as an association of uterovaginal duplication, obstructed hemivagina and ipsilateral renal agenesis [3, 4]. A right-sided prevalence of the obstructed system has been described (as is current case) [4].

Lan Zhu et Al. recommend that the syndrome be classified to two types (classification 1 and 2) according to the complete or partial obstruction of the hemivagina as the clinical details associated with each type are distinctly different [1]. Each of these classifications again has two types:

In classification 1.1, the affected hemivagina is completely obstructed and the uterus behind the septum is totally isolated from the contralateral one. In classification 1.2, the hemivagina is completely obstructed; the cervix behind the septum is maldeveloped/atretic and menses from the uterus behind the septum cannot drain through the atretic cervix. In classification 2.1, there is small communication between the two hemivaginas, which makes the vaginal cavity behind the septum incompletely obstructed. In classification 2.2 the hemivagina is completely obstructed, and a small communication exists between the duplicated cervices (as is in current case). Menses from the uterus behind the septum can outflow through the communication to the offside contralateral cervix. Because the communication is small, the drainage is still impeded [1].

The clinical presentation mainly is related to the type of the abnormality. In classification I, the common clinical presentation can be pelvic pain shortly after menarche, associated with vaginal or pelvic mass [5].

In classification 2, the clinical presentation may be delayed (as in current case), as the obstructed side can be drained through the contralateral vagina. Acute onset of abdominal pain, fever and vomiting can be the presenting symptoms due to bleeding from the fallopian tube [4]. Endometriosis also can occur as a result of blood reflux into the abdominal cavity.

The mainstay of imaging work up is ultrasonography and Magnetic Resonance (MR) imaging. Ultrasonography can show uterovaginal duplication, hematocolpos or hematometrocolpos along with the absence of ipsilateral kidney [5], while MR with multiplanar image acquisition provides more detailed information. It can depict the entire abnormality very well including the presence of communication between the two cervices or vagina (as in current case it could).

In patients with Classification 2.2, hysterosalpingography can also show that contrast material passes through the communication between the duplicated cervices to the contralateral uterus and then the cavity behind the septum [1].

Treatment of choice for obstructed hemivagina is resection of the vaginal septum [6] which can also be performed laparoscopically [7].

Lan Zhu et Al. performed a retrospective long-term follow-up study on surgical prognosis and pregnancy outcomes and found that full resection of the vaginal septum was associated with good outcomes and fertility. No pathologic pregnancies or pregnancy complications were documented [1]. Gholoum S et al. performed a review of 12 pediatric HWWS patients who were treated surgically with vaginal septectomy and drainage of hematocolpos/hematometrocolpos. With median follow-up of 3 years (2 months to 16 years), 11 patients were asymptomatic after treatment and only one patient complained of irregular menses [8].

As the conclusion; HWWS is a rare clinical entity, however it can be suspected in female patients with unilateral absent kidney and pelvic mass. Ultrasonography and MR imaging can well depict the disease entity and surgery is the treatment of choice for obstructed hemivagina. Good long-term outcome is expected after vaginal septectomy.
